# High levels of effective long-distance dispersal may blur ecotypic divergence in a rare terrestrial orchid

**DOI:** 10.1186/1472-6785-14-20

**Published:** 2014-07-07

**Authors:** An Vanden Broeck, Wouter Van Landuyt, Karen Cox, Luc De Bruyn, Ralf Gyselings, Gerard Oostermeijer, Bertille Valentin, Gregor Bozic, Branko Dolinar, Zoltán Illyés, Joachim Mergeay

**Affiliations:** 1 Research Institute for Nature and Forest (INBO), Gaverstraat 4, Geraardsbergen B-9500, Belgium; 2 Research Institute for Nature and Forest (INBO), Kliniekstraat 25, Brussels B-1070, Belgium; 3 Evolutionary Ecology, University of Antwerp, Groenenborgerlaan 171, Antwerpen 2020, Belgium; 4 Instituut voor Biodiversiteit en Ecosysteem Dynamica (IBED), Universiteit van Amsterdam, Postbus 94248, Amsterdam 1090 GE, The Netherlands; 5 Conservatoire Botanique National de Bailleul, Hameau de Haendries, Bailleul F- 59 270, France; 6 Slovenian Forestry Institute, Vecna pot 2, Ljubljana SI-1000, Slovenia; 7 Botanical Society of Slovenia, Izanska cesta 15, Ljubljana SI-1000, Slovenia; 8 8900 Zalaegerszeg, Várberki u. 13, Hungary

## Abstract

**Background:**

Gene flow and adaptive divergence are key aspects of metapopulation dynamics and ecological speciation. Long-distance dispersal is hard to detect and few studies estimate dispersal in combination with adaptive divergence. The aim of this study was to investigate effective long-distance dispersal and adaptive divergence in the fen orchid (*Liparis loeselii* (L.) Rich.). We used amplified fragment length polymorphism (AFLP)-based assignment tests to quantify effective long-distance dispersal at two different regions in Northwest Europe. In addition, genomic divergence between fen orchid populations occupying two distinguishable habitats, wet dune slacks and alkaline fens, was investigated by a genome scan approach at different spatial scales (continental, landscape and regional) and based on 451 AFLP loci.

**Results:**

We expected that different habitats would contribute to strong divergence and restricted gene flow resulting in isolation-by-adaptation. Instead, we found remarkably high levels of effective long-distance seed dispersal and low levels of adaptive divergence. At least 15% of the assigned individuals likely originated from among-population dispersal events with dispersal distances up to 220 km. Six (1.3%) ‘outlier’ loci, potentially reflecting local adaptation to habitat-type, were identified with high statistical support. Of these, only one (0.22%) was a replicated outlier in multiple independent dune-fen population comparisons and thus possibly reflecting truly parallel divergence. Signals of adaptation in response to habitat type were most evident at the scale of individual populations.

**Conclusions:**

The findings of this study suggest that the homogenizing effect of effective long-distance seed dispersal may overwhelm divergent selection associated to habitat type in fen orchids in Northwest Europe.

## Background

Gene flow in plants determines many key aspects of plant ecology including colonization and range expansion, and influences the potential responses to environmental changes. Effective long-distance seed dispersal, (e.g. dispersal followed by establishment) can preserve genetic diversity at the local scale, which may in turn affect the efficiency of selection and local adaptation [1]. Quantifying effective long-distance dispersal (LDD) is therefore crucial to understand whether or not populations are functionally connected, in particular for isolated populations in fragmented habitats. Despite the potential of molecular markers as highly effective tools to study LDD, empirical data on LDD distances in plants are scarce, largely due to the inherent difficulty to identify and sample all the fragments in a given landscape [2]. Species that naturally occur at low densities are particularly suitable for this purpose, as it becomes feasible to map and sample all populations in a landscape.

Most orchid species are typically characterized by small, disjunct populations and are assumed to have a considerable dispersal potential because they produce a huge amount of dust-like, wind-dispersed seeds [3]. Until now, only a handful studies has focused on the spatial aspects of seed dispersal in orchid populations. Evidence from parentage analysis and fine-scale spatial genetic analysis shows that orchid seeds frequently land within metres of the parent plant e.g. [4-6]. However, these studies have focused on short distance dispersal and were not designed to detect the rare long-distance dispersal events that may contribute to colonization and gene flow among populations.

The family Orchidaceae is well known for its exceptional diversity, with approximately 26,000 species. A combination of strong genetic drift and natural selection has been proposed as the key to this immense species diversification [7,8]. A critical requirement of the ‘drift-selection model’ is that effective gene flow is restricted between spatially isolated populations [9]. However, in a meta-analysis of 58 orchid population genetic studies, of which 52 used allozymes, Phillips *et al. *[10] found that orchids are typically characterized by exceptionally low levels of population genetic differentiation (low F_ST_-values) compared to most other plant families. Furthermore, isolation-by-distance was most frequently detected when the scale of the sampling exceeded 250 km, suggesting that below this scale, there is extensive seed dispersal. Phillips *et al. *[10] discussed that drift-mediated speciation is therefore unlikely to be an important mechanism explaining the high diversity of orchids. They argued that LDD combined with local adaptation is likely a possible mechanism underlying the species diversity, but this has not been studied experimentally. Yet, empirical data about effective long distance gene flow and about the proportion of the genome contributing to adaptation and affected by divergent selection is largely lacking.

**Figure 1:**
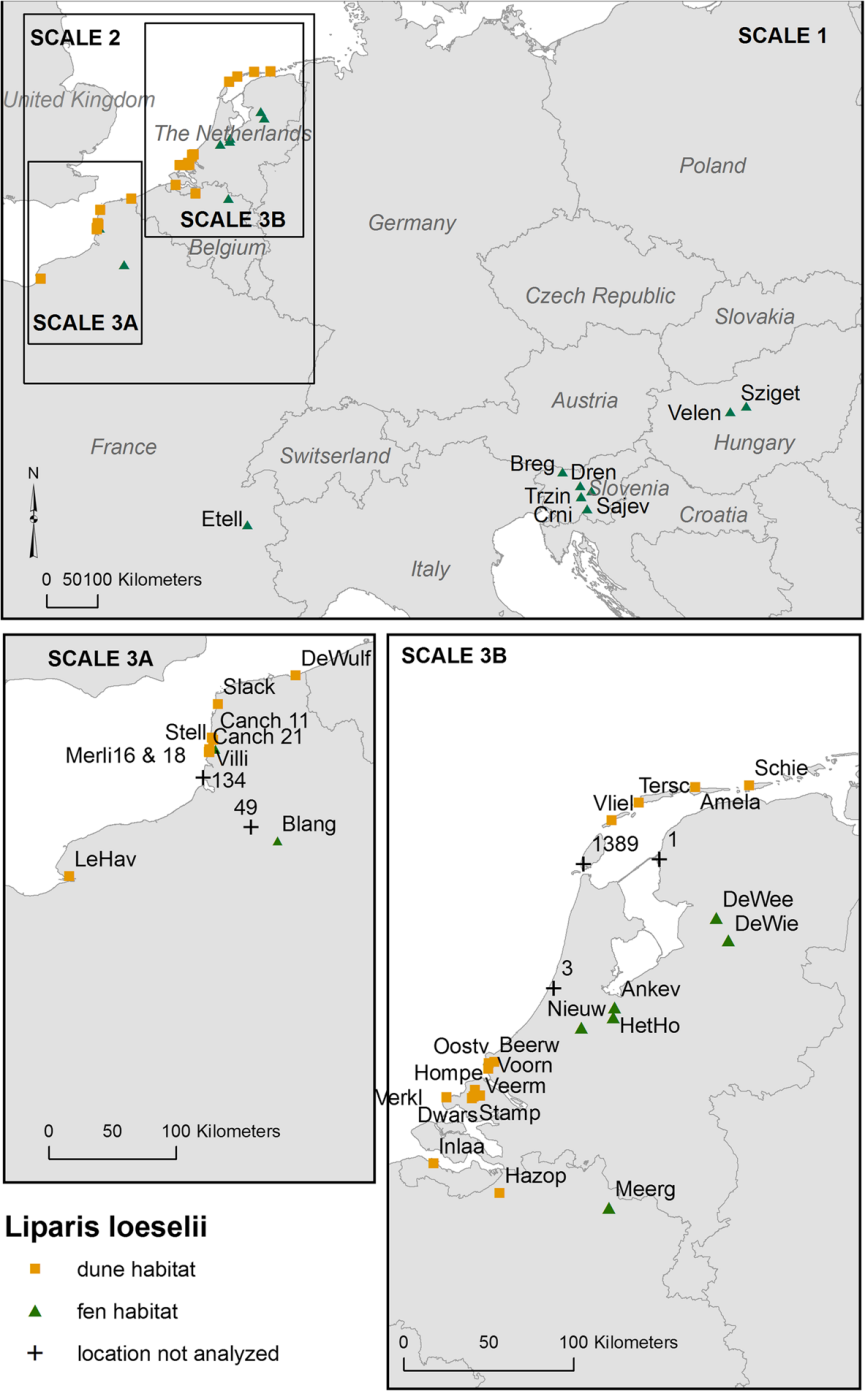
**Map of *Liparis loeselii *** **sampling locations and scales used in the outlier analysis.**

Here, we chose the fen orchid (*Liparis loeselii* (L.) Rich.) to study effective long distance gene flow and adaptive divergence. Fen orchid is a rare species declining throughout its distribution range that covers temperate parts of North-America and Europe. It occurs in early successional vegetation of coastal wet dune slacks and alkaline fens in plains and mountains [11]. As such, it is a typical pioneer plant for which regional metapopulation persistence depends on extinction-colonization dynamics. Within the species, two varieties are sometimes distinguished: a narrow-leaved variety occurring in fens, and a shorter, broader-leaved variety (var. *ovata* Ridd. ex Godfery) occurring in dune slacks [11]. Genetic differentiation may hence exist between the two habitats to the extent that hybrid offspring suffers from marked outbreeding depression (‘isolation by adaptation’, IBA) due to a break-up of co-adapted gene complexes (i.e. immigrant inviability [12]). However, empirical evidence for IBA in plants is scarce (reviewed by Nosil *et al. *[12]) and completely lacking for orchids. Furthermore, understanding the spatial scale of evolutionary processes is required in order to set targets for conservation but little is known about the geographical scale at which local adaptation takes place.The aim of this study was to investigate effective long-distance dispersal by seed as well as adaptive divergence at different spatial scales in the fen orchid. We used AFLP-based assignment tests to quantify long-distance seed dispersal events and their effect on the spatial structuring of genetic diversity across Northwest Europe (Figure 1). By using a genome scan, we looked for loci under divergent selection (outlier loci) related to habitat type to test the hypothesis that IBA contributes to ecotypic divergence. To assess the spatial scale of adaptation, we performed the outlier-analysis at different spatial scales: the continental scale (Europe), the landscape scale (Northwest Europe) and the smaller regional scale (Belgium/the Netherlands and Northwest France). Particularly, we looked for replicated outlier behaviour that would provide evidence of independent and parallel divergent selection.

## Results

### AFLP pattern and genetic diversity

Using four primer combinations we scored 451 polymorphic loci. After excluding samples with low profiles, the remaining total sample consisted of 422 individuals from 38 populations. Information on the sample locations is given in Additional file 1 and in Figure 1. The mean typing error following Bonin *et al. *[13] was 2.4% per locus (see Additional file 2). We observed consistent AFLP-banding patterns and no grouping in the principal coordinate analysis (PCoA) according to the extraction method (results not shown), suggesting no confounding effects of the DNA extraction on the AFLP patterns. A significant negative correlation between fragment sizes and frequencies was found for one primer combination (EcoRI-ACT/MseI-CTA, 250 loci) (*r* = -0.22, *p* < 0.05), which may indicate a potential presence of size homoplasy or suboptimal concentrations in the PCR mix. The exclusion of fragments smaller than 200 bp for this primer combination (73 fragments) resulted in a non-significant correlation (*r* = -0.12, *p* > 0.05). To further reduce potential biases associated with the estimation of population parameters, we further reduced the number of fragments for this primer combination (as recommended by Caballero et al. [14]) from 177 to 65 by excluding all fragments smaller than 350 bp. This resulted in a data subset of 266 polymorphic loci for the four primer combinations. This subset was used to analyse patterns of genetic diversity and genetic structure.

The AFLP band frequency distribution for the 451 polymorphic loci was asymmetric with relatively high occurrences at the low and high frequency ends of the distribution (results not shown). Pairwise logistic regressions between the 451 loci were significant for only 2.47% of all comparisons (*p* < 0.0001), suggesting that less than 3% of all pairwise loci comparisons were not independent. This was further reduced to only 1.0% of significant pairwise loci comparisons (*p* < 0.0001) when repeating the logistic regressions on the subset of 266 loci.

**Figure 2:**
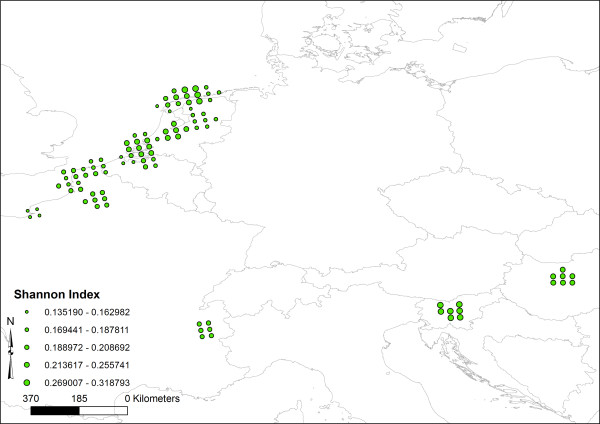
**Regional patterns of genetic diversity of 422 *Liparis loeselii *individuals.** Genetic diversity is calculated by using a sliding window-approach on a 25 km grid (Shannon index, 5 individuals are sampled per grid cell, the displayed results are averaged over 100 bootstraps).

No ramets of the same genet were found among the samples. The estimated selfing rate (*s*) (mean ± SD) calculated over all K subpopulations (for an optimal K = 28) was 91% (±5.5). This corresponds with a mean inbreeding coefficient of 0.83. The proportion polymorphic loci (PPL) at the 5% level, Nei’s gene diversity (H_j_,) and the rarity index (DW-values) are given per population in Additional file 1. The PPL ranged from 32 to 82% with a mean of 59%. H_j_ ranged from 0.13 to 0.35, with a mean of 0.20. Patterns in the unbiased Shannon diversity index are presented in Figure 2. By visual inspection, we detected no clear geographical trend in Shannon diversity.

### Genetic structure

**Figure 3:**
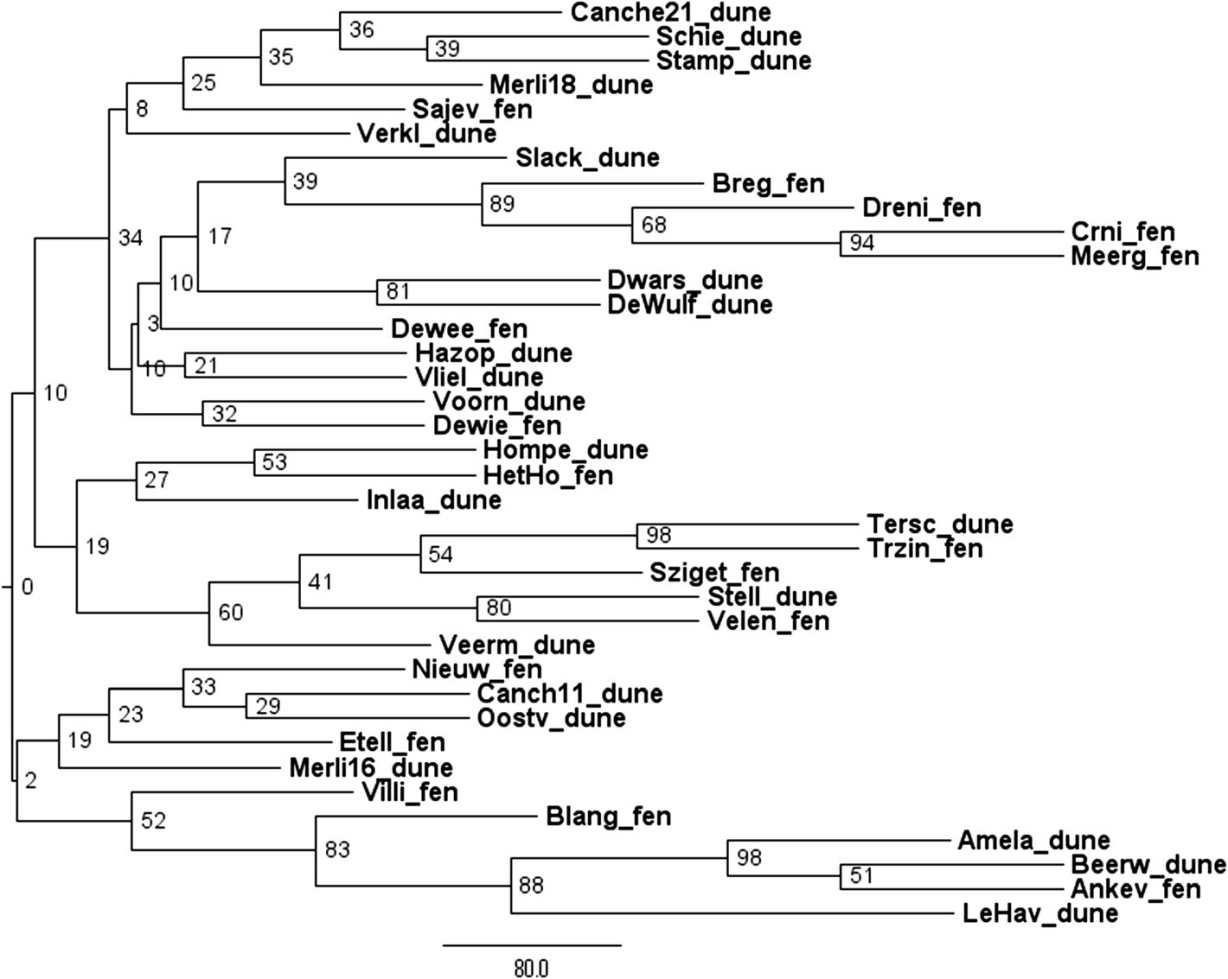
**Midpoint-rooted neighbour-joining tree of 38 *Liparis loeselii *populations calculated from Nei’s genetic distance.** The populations are located in dune slack or fen habitats. The bootstrap support values are based on 100 bootstraps.

**Figure 4:**
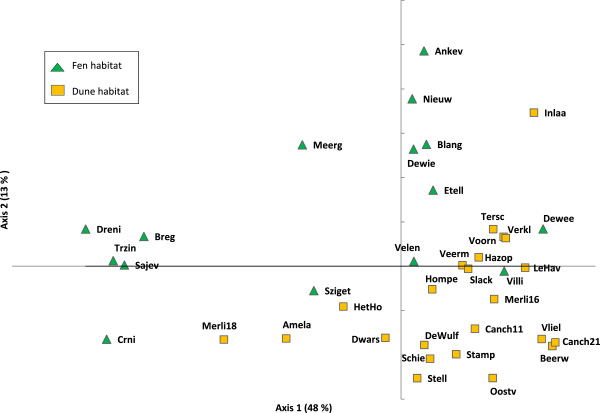
**Principal Coordinates Analysis of pairwise population genetic distances calculated for 38 *Liparis loeselii *** **populations based on 266 polymorphic AFLP markers.**

There was a moderate genetic differentiation between the populations at the continental scale. The F_ST_-value (mean ± SD) calculated for the estimated mean self-fertilisation rate of 91% was 0.09 (±0.1). The mean estimated value of Φ_PT_ was 0.13 (*p* (rand > = data) = 0.001). Clustering at the population level is presented in the Neighbour-Joining (NJ) tree and in the PCoA in Figure 3 and Figure 4, respectively. In general, the NJ-tree showed low bootstrap support and no consistent genetic structure, neither according to geographical location nor to habitat type (fen/dune slack). INSTRUCT indicated the lowest DIC for the model with 28 clusters. Confirmed by the NJ- tree and the PCoA, the Bayesian approach did not group geographically nearby populations consistently within the same genetic cluster and showed a high level of admixture in each population (results not shown). Based on the mean population genetic distances, the PCoA segregated almost completely the populations located in dune-habitats from these located in fen-habitats (Figure 4). However, a PCoA based on pairwise genetic distances between individuals resulted in one large cluster with no segregation of the individuals according to habitat type (results not shown). Extensive gene flow and admixture was also suggested by the absence of a significant isolation-by-distance effect (rxy = -0.015, *p* (rxy-rand > = rxy-data = 0.44)).

### Long distance seed dispersal

The simulations for the assignment procedure resulted in a fairly small increase in proportion of failures at increasing assignment stringency levels. Increasing the latter from a minimum log-likelihood difference (MLD) of 0 (i.e. no likelihood difference threshold between the most likely and the second most likely population) to MLD = 3 (i.e. allocation achieved only if the most likely population is 1000 times more likely than the second most likely population) increased the average rate of failures (i.e. the average rate of wrong allocations combined with the average rate of non-allocations) with 4.2% and 4.5% for the simulated data of Belgium & the Netherlands and Northwest France, respectively. Consequently, our AFLP-dataset proved to be adequately powerful. The average estimated rates of allocation success, of non-allocation and of allocations to the wrong population for different values of MLD and based on the simulated datasets (10 iterations × 1000 genotypes) are given in Additional file 3. The re-allocation results and the effect of the number of putative source populations and of the number of loci on these results are given in Additional file 4.

**Figure 5:**
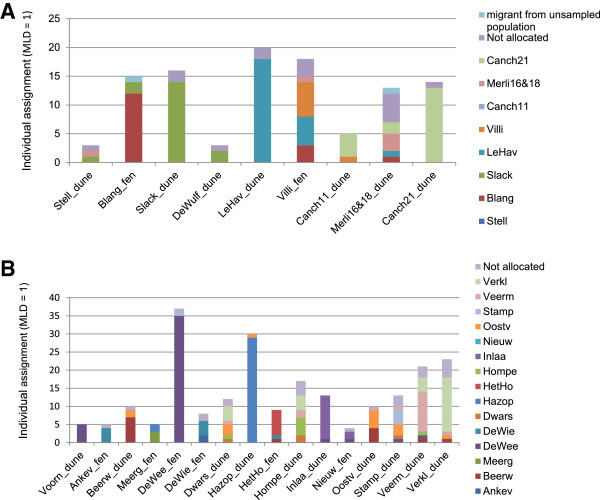
**Individual assignment of individuals of *Liparis loeselii *sampled in Belgium & the Netherlands (A) and Northwest France (B).** Results obtained with AFLPOP under minimal log-likelihood difference (MLD) set to 1 and based on 451 polymorphic AFLP markers.

For Northwest France, two clusters of two redundant loci each were found and reduced to a single locus. The re-allocation tests on this reduced dataset identified 24 putative migration events within Northwest France representing 12 different source – destination combinations (13.0%). Another two individuals (2.2%) were allocated to a source population that was not sampled. This resulted in an estimate of the LDD rate between 15.2% and 28.2% for the sampled populations in Northwest France. The simulation analysis for the populations of Northwest France resulted in 71.5% correct allocations. This increased to a success rate of 99.9% (*p* = 0.001) when excluding locations with low sample size (n ≤ 5). Dispersal distances ranged from 1.95 to 152 km with a median geographical distance between the different combinations of source- destination populations of 50.6 km. When excluding locations with a low sample size (n ≤ 5), we obtained an estimate of the LDD rate between 9.3 and 17.7% and a mean dispersal distance of 74.8 km (range: 4.9 – 152). The main directions of LDD were northwest and northeast, each representing 33% (28%, after excluding locations with n ≤ 5) of all the different source – destination combinations. Re-allocation tests suggested seed dispersal between populations occupying different habitats (Figure 5).

For the populations of Belgium and the Netherlands, no clusters of redundant loci were detected. Within this region, 61 putative migrants were identified, representing 32 (16.5%) different source – destination combinations. No putative immigrants from outside the sampled region were detected. This resulted in an estimate of LDD for the populations of Belgium and the Netherlands ranging from 16.5 to 30.5%. Simulation tests estimated an allocation success rate of 94.9% (*p* = 0.05). LDD distances ranged from 1.64 to 220.7 km with a median geographical distance between the different combinations of source – destination population of 20 km. The main direction of LDD was southwest which represented 43% (14 out of 32) of the different source – destination combinations. Also at this regional scale, assignment tests suggested seed dispersal between populations occupying different habitats (Figure 5).

For the re-allocation procedure of the samples from Belgium and the Netherlands, including the populations from Northwest France as putative source populations changed the assignment for four out of 61 (6%) putative immigrants from a population from Belgium/the Netherlands to a population located in Northwest France. Including the four sampled populations on the Dutch Wadden islands as putative source populations within the re-allocation tests resulted in a different assignment for three putative migrants (5%). For the re-allocation procedure of the samples from Northwest France, a higher number of putative source populations changed the assignment for seven out of 24 allocated individuals (29%) from a neighbouring French population to a population located in Belgium/the Netherlands. Removing loci that have a high probability of being homoplasious (73 loci) from the dataset increased the number of individuals that could not be allocated (see Additional file 4) and changed the assignment of five (2.5%) and two (2.2%) allocated individuals for the regions of Belgium/the Netherlands and Northwest France, respectively.

### Putative adaptive loci

One locus (outlier ID 167 (ACTcta148)) was identified as an outlier associated with habitat-type by both BAYESCAN and MCHEZA at the continental scale (scale 1) (Table 1). However, this locus was not retained as a significant outlier as it emerged as such in one pairwise (control) fen-fen population comparison (between Blang and Dewee (see Additional file 5)). No outlier loci were detected by both BAYESCAN and MCHEZA in the overall between-habitat comparisons at the landscape and at the regional scale.

**Table 1:** **Results of the outlier analysis for directional selection of AFLP loci in the overall comparison between dune and fen habitats of *Liparis loeselii***

**Geographical scale**		**No. of dune samples**	**No. of fen samples**	**No. of outliers (BAYESCAN; MCHEZA)**	**Common outliers**	**Outlier ID BAYESCAN**^**1 **^**(P(α ≠ 0))**	**Outlier ID MCHEZA**^**2**^
Continental (1)	total data	273	117	1; 1	1	167 (0.97)	167
Landscape (2)	B, NL, NW- F	273	101	2; 2	0	179 (0.93), 446 (0.98)	167, 444
Regional (3a)	NW-F	74	33	0; 15	0	-	146, 167, 467, 178, 259, 294, 312, 418, 422, 429, 434, 450, 254, 444, 437
Regional (3b)	B, NL	199	68	3; 2	0	162 (0.99), 164 (0.99), 446 (0.98)	167, 444

Populations sharing the same habitat-type were pooled on different geographical scales. B: Belgium; NL: the Netherlands; NW-F: Northwest France.

^1^Locus detected as significant outlier locus using a threshold of posterior odds (PO) >10 or P(α ≠ 0) > 0.91; 'strong evidence' for selection.

^2^The F_ST_ cut-off value for significant outlier detection was set to 0.99.

Six loci (1.3%) were identified as outliers in at least one pairwise fen-dune comparison and not in the control fen-fen and dune-dune comparisons (see Additional file 5). These loci (ID 157 (ACTcta138), ID 163 (ACTcta143), ID 368 (ACTcac376), ID 410 (ACTcta448), ID 431 (ACTcta85) and ID 440 (ACTcta91)) were identified as outliers with ‘strong evidence’ (*p* (α > 0.91)) in BAYESCAN. Of these, only one locus (0.2%) (ID 431) was a replicated outlier in three multiple pairwise population comparisons of which two were statistically independent, that is, comparisons that did not share a population. These comparisons included two different fen populations from the Netherlands (Dewee and the pooled sample HetHo, Nieuw & Ankev) and three different dune-populations (1/Canch11 & Canch21; 2/Merli16 & Merli18 & Stell and 3/Tersc (see Additional file 5).

## Discussion

### High levels of effective long-distance dispersal

This study suggests remarkably high levels of inter-population seed dispersal in fen orchids in Northwest Europe. Given its autogamous pollination system, gene flow by pollen is likely to be negligible [15], as also shown by our estimate of the selfing rate (91%), and thus the species primarily disperses its genes by seed. At least 15% of the assigned individuals likely originated from among-population seed dispersal events with dispersal distances up to 220 km. Only 61.2% of all sampled individuals were assigned based on genotype to the population from which they were sampled and 11% remained unassigned. After relaxing the criteria of assignment, all of these unassigned individuals seemed to originate from the populations within which they were sampled. Insufficient genetic resolution between the source population and one or more unsampled source populations may be the reason for these unassigned individuals [16]. In many cases, the dispersal events observed did not occur between adjacent populations. For the region of Belgium and the Netherlands, the main dispersal direction followed the second predominant wind direction, after dominant overseas western winds, with 43% of the different putative source – destination dispersal events coming from the southwest. For the region of Northwest France where overseas west to southwest winds are predominant, the main source – destination dispersal directions were northeast (the second predominant wind direction), and northwest. Assignment success was high for the samples from Belgium and the Netherlands (assignment success: 90%, probability of correct assignment: 94.5%) but lower for the samples of Northwest France (86%, probability of correct assignment: 71.5%) likely because of the low sample sizes (n ≤ 5) for three locations. Excluding these latter locations from the assignment analysis increased the probability of correct assignment, calculated based on the simulations, to 99.9% and decreased the lower bound of the LDD-estimate for Northwest France from 15% to 9%. Enlarging the geographical scale by including more putative source populations had more influence on the allocation of putative migrants from Northwest France (seven migrants (29%)) compared to the allocation of putative migrants from Belgium and the Netherlands (four migrants (6%)), which is in accordance with the estimated probability of correct assignment.

Orchids produce thousands to millions of extremely small (<0.5 mm) seeds per capsule [3]. Their seeds have large internal air spaces that make them balloon-like and facilitate LDD [3]. These seed characteristics combined with the high fecundity likely explain the LDD events observed in this study. The maximum distance of seed dispersal of 220 km could only have been detected by studying an area of this size, illustrating the limited value of estimates of average dispersal distances derived from spatially restricted studies. Given the frequent strong coastal winds, it is likely that the maximum distance of seed dispersal is even much higher than the distances observed here. Indirect methods based on dispersal models indicate dispersal capabilities of orchid seeds by wind of up to 2000 km [3]. High levels of LDD will likely reduce the probability that seeds will reach a suitable habitat, as many seeds are ‘lost’ in the unsuitable matrix between the habitat patches [17]. However, LDD is needed to transport seeds from local, high-competition patches to remote, low-competition patches, and thus enhance seedling survival [17]. The results of extensive seed dispersal are consistent with the observed absence of a significant relationship between genetic and geographic distance and a moderate genetic differentiation among populations (F_ST_ = 0.09, Φ_PT_ = 0.13). The F_ST_-values observed in this study confirm the rather low F_ST_ in orchids compared to other herbaceous families (reviewed by Phillips *et al. *[10]). The absence of a structure in the genetic data according to the geographic location was also reported by Pillon *et al.*[18] in a study on the fen orchid in Northwest France and the United Kingdom. Being a pioneer and predominantly selfing species, the fen orchid generally colonizes an open habitat with one or a few individuals, and subsequent population expansion mainly results from the establishment of progeny of the original founders. Seeds that act as founders in unoccupied patches, have a subdividing effect which results in an increase of F_ST_ [17]. In contrast, long-distance dispersed seeds arriving at already occupied patches have a homogenizing effect on the genetic structure of the metapopulation, thereby decreasing F_ST_ [17]. The moderate value for F_ST_ and the observed large rates of long-distance seed dispersal among established populations suggest that the homogenising effect of gene flow is stronger than the subdividing effect of founder events for fen orchid in Northwest Europe. This post-colonization gene flow has also been shown to be important as a ‘rescue effect’ at the metapopulation level [19]. Under low extinction probabilities, the homogenizing effect prevails whereas the subdividing effect dominates at intermediate to high extinction probabilities, especially in expanding populations [17]. At intermediate levels of local extinction, LDD clearly raises metapopulation survival as compared to short distance dispersal [17,20]. Local populations of the fen orchid are generally assumed to have high extinction probabilities but empirical data on local population lifetimes are largely lacking. The homogenising effect of gene flow indicates that at least some populations may not be particularly young. Indeed, the presence of the fen orchid population at the Belgian location Meergoor is documented since 1975 and this population appears to persist on this location for over 45 years [21]. Yearly observations of fen orchid individuals on the same location over a time span of seven and eight years were reported for the French fen population Le marais de Pagny-sur-Meuse [22] (located in northeast France, not included in this study) and the Belgian population Hazop (unpublished data), respectively, indicating population lifetimes of at least seven years. Whether the fen orchid forms a seed bank is not known but terrestrial orchid seeds are generally short-lived (1 to 5 years) [23]. It is therefore unlikely that some of the fen orchids assigned to a distant population may have actually originated from a local, long-lived seed bank.

The results of this study also indicate high admixture between populations of fen and dune slack habitats. Remarkable for a predominantly selfing species, we observed substantial genetic diversity within populations (mean PPL: 59%). A high level of AFLP polymorphism, the absence of identical genotypes, a similar asymmetric AFLP band frequency distribution and a relatively low level of linkage disequilibrium were also found for *Arabidopsis thaliana,* which, as the fen orchid, reproduces almost exclusively through selfing [24]. Miyashita *et al.* (1999) explain this nucleotide polymorphism by recombination events and random mutations. Comparable to this study, relative high population genetic diversity values (H_j_., range: 0.13 - 0.24, mean: 0.18) for the adult stage were also found in the predominantly autogamous food-deceptive orchid *Neotinea maculata* using AFLP loci [25]*.*

Previous studies have shown that AFLPs were efficient in assigning each individual to its population, especially at intermediate spatial scales and when population differentiation is weak [16,26]. The power of AFLP-based assignments increases with the number and quality of the AFLP-loci with low-informative loci (i.e. loci with allele frequencies close to 0 and 1) strongly contributing to the assignment power [26]. In this study we used a large number of loci and many were low-informative, indicating a broad genome coverage and a good assignment success. However, for some populations the number of plants analysed was small. Assignment tests assume that allele frequency estimates are accurate. Although fen orchid is known to be subject to strong founder effects, selfing populations may show variable genetic diversity depending on the number of selfing-lineages. It is therefore possible that we have missed selfing-lineages because of small sample sizes. This may have affected the accuracy of the allele frequency estimates. Furthermore, for old populations some dispersal events may be several generations old and may have originated from a population that currently has become extinct. Such events may have resulted in an overestimation of the seed dispersal distances. This may be the case for some populations located in relative stable fen habitats. Yet, many dune-populations studied here are known to be relatively young, resulting from colonisation events that have occurred in the last few decades when fen orchid was already rare in the study area [21,22]. Still, the above estimates of effective long-distance seed dispersal should be treated cautiously. Though this study suggests extensive gene flow in fen orchid, the actual connectivity of populations may be lower than the estimated dispersal distances reported here suggest.

### Signals of adaptive divergence

To date, few studies exist on the spatial scale of local adaptation at the genetic level [but see [27]. Here, we tested for significant differentiation associated with habitat type at different geographical scales. When pooling populations sharing the same habitat at different geographical scales one locus (ID 167) was identified as a common outlier at the continental scale including all the sampled populations, by both BAYESCAN and MCHEZA. This locus was however also identified as an outlier in one pairwise comparison of two fen populations. Therefore, we did not consider ID 167 to be associated with divergence between habitat types. Similar to the continental scale, no reliable outliers were detected when pooling populations at the landscape and the regional scale. However, at the level of individual populations, we observed six outlier loci in at least one pairwise population comparison potentially reflecting a signature of adaptive divergence associated with habitat type. These loci were identified as outliers in pairwise among-habitat comparisons and not in control comparisons. Of these loci, ID 431 was an outlier in two statistically independent pairwise population comparisons, suggesting replicated divergence. The latter is unlikely to arise via non-selective factors such as type I error, genetic drift or mutation rate variation and is therefore a powerful application of the genome scan [12]. This may demonstrate the repeated and parallel fixation of the same adaptive allele and suggests that some fen orchid populations may have locally adapted to habitat type. Adapted populations may have evolved preferences for their native habitat, which could have decreased effective dispersal and mating between-habitats and lowered the viability of immigrants (i.e. IBA). Hence, these findings may suggest that local adaptation to habitat type in the fen orchid is more likely to occur at the level of individual populations rather than at larger geographical scales. This is consistent with metapopulation genetic theory which predicts, under the island model, that founder effects associated with patch colonization play the primary role in creating genetic divergence among local populations [1]. Divergent adaptation may proceed via different mutations in different localities such that particular outliers are not highly consistently observed across population comparisons [12]. LDD may enhance speciation at moderate colonization-extinction rates. If local extinction is absent or too frequent, the genetic homogenizing effect of LDD will prevent speciation [17]. It is also possible, however, that the outliers identified are related to any other kind of selection and not necessarily to divergent selection associated with habitat type [28]. The importance and function of the detected outlier loci and their neighbouring genes for adaptation remain to be clarified in future experiments. Although we detected a few outliers that potentially reflect adaptive divergence, we did not find strong signals of IBA. Other studies examining genomic divergence in plants, report 0.4 to 35.5% outliers (reviewed by Strasburg *et al. *[29]). But, as Nosil *et al. *[12] pointed out, comparisons across studies are difficult because of the variety of analytical approaches and of the range of significance cut-offs used by different researchers.

There are several possible explanations for the lack of strong signals for adaptive divergence associated with habitat type in the fen orchid. One possible explanation is that the levels of genetic adaptive divergence across the genome are too low to be detected by a genome scan. If divergent adaptation occurs through moderate changes in allele frequencies at multiple sites, it is likely that none of these sites will exhibit substantial divergence between populations [28]. Besides this limitation of genome scans, gene flow combined with the selection strength and the timescale may explain the lack of strong signals of IBA. Adaptive divergence between populations will only occur if reproductive barriers are strong enough to restrict gene flow at ecologically relevant loci [12]. The findings of this study indicate high levels of effective gene flow in the fen orchid over long distances, also between populations occupying different habitat types. It is thus plausible that the homogenizing effect of effective gene flow overwhelms the signals of divergent selection and thereby mostly erases the signal of IBA [30]. In addition to high levels of gene flow and selection strength, the short life span of individuals and the high turn-over rate of populations result in a short timescale for diversifying selection to act on allele frequencies in favour of one or the other habitat type, which may also explain the observed low signals of IBA in fen orchids.

## Conclusions

Founding effects from long-distance seed dispersal combined with local adaptation to regional variation, rather than drift-mediated selection, have been proposed by Phillips *et al. *[10] as key factors in the diversification of the Orchidaceae. The results of this study support this hypothesis but also suggest that high levels of effective gene flow may strongly act against speciation by erasing differences developing between populations. However, founder effect speciation by reproductive isolation may evolve over several hundred generations. It is possible that we detect no strong signals of IBA because the fen orchid may currently be in an early stage of the process of population differentiation and speciation. To further investigate ecologically important functional variation, genomic data should be combined with fitness characteristics and morphological data from reciprocal transplant experiments with individuals originating from different habitats.

## Methods

### Study species

Fen orchid (*Liparis loeselii* (L.) Rich.) is a small, diploid (2n = 26) orchid that relies on regular disturbance for its survival [31]. This species perenniates during winter and the leaves appear above the ground in mid-June to mid-July [11]. Flowering occurs from late June to mid-July. The inflorescence has 2 to 23 small, yellow-green flowers that are apparently nectarless and scentless [31]. Like most other orchid species, the fen orchid is self-compatible. Observations of insect visitors are extremely rare [15,31]. It appears that the fen orchid is predominantly autogamous, with self-pollination facilitated by rain-drops [15]. Fruit capsules ripen by mid-October. Fen orchid produces thousands of wind-dispersed seeds per capsule [3]. Viability of seeds is high (60% to 97.2%) [6 counts in [31]]. Whether the fen orchid forms a seed bank is unknown but terrestrial orchid seeds are generally short-lived (lasting 1 to 5 years) although some species may be capable to form a seed bank that last almost 7 years [23]. Genets may flower for two or more consecutive years or may remain several years in vegetative state but pseudobulb dormancy is unlikely to occur in this species [11]. Genets are short-lived (2-3 years) and populations are characterized by a high turnover-rate [11].

### Plant material

Because of the high conservation priority in Europe, the fen orchid is extensively surveyed since 1992 (implementation of Habitat Directive (92/43/EEC)) and the localities of existing populations in France, Belgium and the Netherlands are well known [21,22,32]. This allowed us to identify the vast majority if not all locations of this species along the coast of the North Sea and on more inland locations over a distance of 600 km from Normandy in Northern France up to the Dutch Wadden Sea islands in the north of the Netherlands. Adult plants were sampled from 32 out of 36 known locations (Figure 1). The remaining four locations were not included because of logistic sampling difficulties. Furthermore, we sampled one population in the French Alps in the Marsh of Les Etelles, five populations in Slovenia located in the pre-alpine hills and North-western Dinaric Mountains, and two populations in Hungary: one at Lake Velence and one at Szigetcsép. In total 718 plants from 39 locations representing 23 dune slack and 16 fen populations were attempted to be analysed. Eleven locations were sampled during summer 2003, the rest in summer 2009. The census population sizes were widely distributed, ranging from a few to thousands of individuals (Additional file 1). The number of individuals sampled per population varied from 3 to 72 and depended on the census population size, the accessibility of the locations and the permits of sampling. In large populations, plants were sampled randomly with a minimum distance of one meter between sampled plants. Sampling permits were obtained from relevant authorities. No more than half a leaf was collected from adult plants. This sampling has no marked deleterious effect on the plants [18].

### DNA extraction and AFLP analysis

Total DNA was extracted from silica dried leaf samples with the QuickPickTM Plant DNA kit (Isogen Life Science, De Meern, the Netherlands), except for the samples collected in 2003 for which the CTAB-based method [33] was used. AFLP-fingerprints were generated according to Vos *et al. *[34], with restriction and ligation conducted in one single step. After preliminary tests, four primer combinations were chosen which resulted in clear bands of sufficient variability (see Additional file 2). PCR products from each primer pair were run on an ABI 3500 capillary sequencer (Applied Biosystems). Genescan 600-Liz (PE Applied Biosystems) was used as an internal lane size standard. Only samples with high-quality AFLP profiles and fully genotyped for the four primer combinations were retained for further analyses. Raw data were sized with GeneMapper 4.1 (Applied Biosystems). To test for reproducibility, 40 samples were randomly chosen from the samples collected in 2009 and replicated from the DNA-extraction step. A binary matrix of AFLP band presence (1) – absence (0) was built using the automated scoring package RawGeno v 2.0 (R CRAN; Arrigo *et al. *[35]) using the scoring parameters: MINBIN = 1, MAXBIN = 2, FREQ = 1, THRESH = 80. The range of fragments scored for each primer-enzyme combination is given in the Additional file 2. The replicated samples allowed the removing of non-reproducible bins and subsequently, the calculating of the error rate according to Bonin *et al. *[13] with RawGeno v 2.0. We checked the consistency of the AFLP-banding patterns over the two different DNA-extraction methods with GeneMapper 4.1 by visually comparing the overall AFLP-profiles and the position of the monomorphic loci over the two extraction methods. To further check for possible AFLP-artefacts, we performed a PCoA using GenAlEx 6.5 [36] with the samples labelled according their extraction method. As recommended by Vekemans *et al. *[37], the correlation between AFLP band size and frequency among samples was assessed for each primer combination to check for potential homoplasy. Linkage disequilibrium among AFLP loci was tested using pairwise logistic regressions [e.g. 21]. We used the false discovery rate (FDR) based multiple comparison procedure [38] to correct for multiple testing. The maximum FDR was set to 5%. The calculations were performed using the R packages fdrtool 1.2.10 [39] and brainwaver 1.5 (http://cran.r-project.org/web/packages/brainwaver/). Before performing further analysis, we excluded loci with frequencies below 5% and above 95% that may lead to spurious correlations and are therefore not considered reliable [40].

### Patterns of genetic diversity

As the fen orchid can reproduce vegetatively, we first checked whether the dataset contained similar ramets of the same genet with AFLPDAT [41] by setting the maximum number of differences among identical individuals to the calculated error rate. The self-fertilisation rate (s) was estimated at the 0.95 significance level with the program INSTRUCT [42]. Allele frequencies were estimated with AFLP-SURV v 1.0 [37] using a Bayesian approach and a non-uniform prior distribution of allele frequencies and an inbreeding coefficient calculated from the estimated s (F_IS_ = s/(2-s)). Genetic diversity was investigated by computing the proportion of polymorphic loci (PPL) at the 5% level and Nei’s gene diversity (Hj, analogous to He) using standardized sample sizes (i.e. with a maximum of eight samples per population). Frequency down-weighted marker values (DW-values or rarity index) were calculated with AFLPDAT [41].

Spatial patterns of genetic diversity were inferred based on the Shannon index using a geographical ‘sliding window’ approach as in Arrigo *et al. *[43]. The analysis considers a 25 km grid over the whole sampling area and computes the Shannon index by considering samples located within a radius of 35 km around each grid point. In order to provide an unbiased Shannon index under unequal sampling among areas, computations were bootstrapped by a 100 times resampling with 5 samples per grid point. Computations were performed using R scripts (R Development Core Team, 2009, script obtained from Nils Arrigo).

### Genetic structure

As a measure of population differentiation we calculated F_ST_ using AFLP-SURV1.0 with 100 permutations and assuming the selfing rate estimated from the data by INSTRUCT. In addition, pairwise Φ_PT_ were estimated using the AMOVA analysis of GenAlEx 6.5 [36]. The significance of each value was calculated using the Monte Carlo procedure (999 permutations). The genetic structure of populations was investigated using several clustering approaches. With GenAlEx 6.5 we computed a matrix of pairwise population genetic distances [44] on which we performed a PCoA. Nei’s pairwise population genetic distance matrices calculated by AFLP-SURV v 1.0 and assuming the same inbreeding coefficient as above, were used in the NEIGHBOR and CONSENSE procedures of the package PHYLIP v 3.69 [45] to obtain consensus neighbour-joining trees. The program FIGTREE 1.3.1 (http://tree.bio.ed.ac.uk/software/figtree/) was used to display the midpoint rooted dendrogram.

Furthermore, a Bayesian clustering approach implemented in INSTRUCT [42] was run under the population-specific model with admixture. We ran four parallel MCMC chains on four different processors for each assumed number of clusters K, with K set from one to 38. Each chain contained 200 000 iteration steps, 100 000 burn-in iterations, and a thinning interval of 20 steps, assuming different starting points. For each chain, the optimal K was inferred based on lowest value of the Deviance Information Criterion [46]. To check for isolation-by-distance, a Mantel test between pairwise Φ_PT_ and pairwise geographic distances was performed as implemented in GenAlEx 6.5 (999 permutations).

### Individual assignment tests

Population likelihood assignment tests for individuals based on their multi-locus genotype were used to estimate contemporary, effective seed dispersal events. We followed the procedure of Duchesne & Bernatchez [47], developed for AFLP markers and implemented in AFLPOP v.1.1. This approach is based solely upon AFLP band frequencies and the assumptions that frequency estimates per population are accurate and that the loci are statistically independent (no linkage disequilibrium). AFLPOP infers for a given genotype and a set of sampled populations, the most likely source population. One major advantage of the method is that populations do not have to be sampled exhaustively [26]. We first used the loci filtering procedure to reduce clusters of redundant loci (potentially linked) to a single locus. A minimum log-likelihood difference (MLD) of 1 was used to assign specimens to the most likely population (re-allocation procedure). This means that a genotype has to be ten times more likely to be found in a given population than in any other population in order to be assigned to that population. In case MLD’s are smaller than one, individuals could not be assigned unambiguously to one of the sampled populations. Prior to the assignment tests, we assessed the statistical power of the dataset for accurate assignment success with the assignment simulator of AFLPOP 1.1. The simulator generated 1000 genotypes randomly from each population based on the observed allele frequencies. Next, the simulated genotypes were re-assigned to the most probable population. These simulations were conducted at four different stringency levels (MLD = 0, MLD = 1, MLD = 2 and MLD = 3) and repeated 10 times to check for the consistency of the results. Increasing values of stringency will decrease the rate of wrong allocations but will also increase the rate of non-allocated individuals. Based on the simulated genotypes, average estimated rates of allocation success, of wrong allocations and of non-allocations were calculated with the simulation procedure of AFLPOP 1.1. The rationale in using different stringency levels is to evaluate the discriminating power of a set of loci. The higher the assignment success at high levels of stringency, the higher the discriminating power of the set of loci will be [26].

Taking into account impacts of founding events (in principle one individual can establish a new population), the number of different source (seed population) – destination (sampled population) combinations where source and destination are not identical, was considered as the lower bound of the estimated seed migration rate. The proportion of putative immigrants among the total of unambiguously assigned individuals was considered as the upper bound of the estimated migration rate. Seed dispersal distance and direction were inferred for each putative immigrant.

Assignment tests were performed within two independent sites in which we are quite certain to have allocated all the fen orchid populations: i) Northwest France and ii) Belgium and the Netherlands with the populations on the Dutch Wadden Sea Islands excluded because of missing data for one large population of the Dutch Wadden Sea Islands (Figure 1). This allowed us to obtain replicated data on LDD. The maximum distance between populations within the sampled regions was 211 km for Northwest France and 220 km for Belgium and the Netherlands. We calculated a p-value for each individual’s log-likelihood by creating empirical distributions from 1000 randomly generated genotypes based on the presence frequencies of each population. When the p-values for an individual were below a certain warning threshold (p < 0.001) for all candidate populations, it was concluded that the individual did not originate from any of the analysed populations [26]. As a result, the individuals with p-values below the threshold for all candidate populations are putative migrants that likely originate from outside the sampling region or from the few locations that were not analysed (Figure 1). To test the potential effect of the number of putative source populations on the assignment of putative immigrants, we repeated the re-allocation procedure within the whole region of Northwest France, Belgium and the Netherlands including 25 source populations (again, populations on the Dutch Wadden Sea Islands excluded). The potential effect of the exclusion of the Dutch Wadden Sea Island populations was also tested by performing the re-allocation procedure for the samples of Belgium and the Netherlands with the four sampled populations on the Dutch Wadden Islands included as putative source populations (16 + 4 source populations). In addition, we tested the effect of the number of loci on the assignment results by comparing the results based on the total dataset with the results based on a subset excluding fragments that showed a high probability of being homoplasious (fragments <200 bp for primer combination EcoRI-ACT/MseI-CTA).

### Detection of outlier loci

In comparison with expectations for neutral evolution, marker loci with excess differentiation are considered to indicate candidate regions under divergent selection. Outlier loci display unusually high values of F_ST_ by comparing observed F_ST_-values with values expected under neutrality. Outlier locus detection was performed based on 451 polymorphic loci by two commonly employed approaches implemented by the programs MCHEZA [48] and BAYESCAN 2.01 [49]. A detailed description on the search of putative outlier loci is given in the Additional file 6. Overall between-habitat comparisons were conducted at three geographical levels, pooling populations of the same habitat-type (Figure 1); 1) at the continental scale encompassing all analysed populations (scale 1: ndune = 273, nfen = 117), 2) at the landscape scale including the populations from the Netherlands, Belgium and Northwest France (scale 2: ndune = 273, nfen = 101) and 3) at the smaller regional spatial scale within Northwest France (scale 3a: ndune = 74, nfen = 33) and within Belgium and the Netherlands (scale 3b: ndune = 199, nfen = 68), respectively. In addition to the overall between-habitat comparisons, we tested for outliers in all possible pairwise population comparisons at the regional level (Figure 1, scale 2). This approach allowed us to assess if loci were predominantly outliers at the continental or the regional scale, or in specific among population pairwise comparisons.

Although DFDIST was shown less reliable than BAYESCAN with AFLP data [50], we expected that a strong signal in the data should be picked up by both methods, making these parallel analyses worthwhile [see also [51]]. MCHEZA and BAYESCAN were therefore both used for the overall between-habitat analysis (geographical scales 1, 2, 3a and 3b) which uses the largest sample sizes and provided the highest power to the analysis. Pairwise comparisons between individual populations were performed with BAYESCAN.

We defined a locus as a significant outlier if it was detected by both MCHEZA and BAYESCAN in at least one of the overall between-habitat comparisons of the pooled population samples (continental, landscape or regional scale) or if it was detected by BAYESCAN in at least one population pairwise comparison in which the pair of populations were located on different habitats. The significant outlier was not retained if it also occurred as an outlier in comparisons between populations of the same habitat type (control comparisons). We defined replicated outliers as outliers associated with different habitats in at least two different, statistically independent pairwise population comparisons thus providing evidence for truly ‘parallel’ divergence.

### Availability of supporting data

The data set supporting the results of this article is available in the Dryad repository

Vanden Broeck, A., Van Landuyt, W., Cox, K., De Bruyn, L., Gyselings, R., Oostermeijer, G., Valentin, B., Bozic, G., Dolinar, B., Illyés, Z., Mergeay, J. (2014): AFLP data from: **High levels of effective long-distance dispersal may blur ecotypic divergence in a rare terrestrial orchid.***Dryad*. doi: 10.5061/dryad.sb68v http://doi.org/10.5061/dryad.sb68v.

## Competing interests

The authors declare that they have no competing interests.

## Authors’ contributions

AV, WV, RG, LD, GO and JM designed the research, AV, WV, RG, GO, BV, GB, BD and IZ generated the data, AV and WV performed the research, AV, KC, RG analyzed the data, AV wrote the paper and all authors commented on multiple drafts of the manuscript. All authors read and approved the final manuscript.

## Supplementary Material

Additional file 1 **Description of the 38 sampling locations of *Liparis loeselii*.** Click here for file

Additional file 2 **Number of DNA fragments generated by 4 AFLP primer-enzyme combinations used in *Liparis loeselii.*** Click here for file

Additional file 3 Effect of different values of the minimal log likelihood difference (MLD) on the estimated rates of allocation success, of allocations to the wrong population and of non-allocations calculated with the simulation procedure of AFLPOP 1.1 and based on 1000 simulated genotypes and 10 iterations. Click here for file

Additional file 4 **Re-allocation results of *Liparis loeselii *individuals performed with AFLPOP 1.1 for a minimal log likelihood difference (MLD) = 1.** Click here for file

Additional file 5 **Posterior probabilities for the model including selection of putative outlier loci detected with BAYESCAN 2.01 in pairwise population comparisons of *Liparis loeselii.*** Click here for file

Additional file 6 Detailed description on the search of putative outlier loci. Click here for file
