# Supplementary material for: High levels of effective long-distance dispersal may blur ecotypic divergence in a rare terrestrial orchid
Source: BMC Ecol. 2014 Jul 7;14:20. doi: 10.1186/1472-6785-14-20 (PMC4099500; doi:10.1186/1472-6785-14-20)
Supplement: Additional file 1 — Description of the 38 sampling locations of Liparis loeselii. [file 1472-6785-14-20-S1.docx]

# Additional file 1

**Description of the 38 sampling locations of Liparis loeselii.**

| Country | Location | Code | Ecotype | Nc | N | DW | PPL^1^ | Hj^1,2^ |
| --- | --- | --- | --- | --- | --- | --- | --- | --- |
| the Netherlands | Schiermonnikoog, Dutch Wadden sea | Schie | dune | 108 (2003) | 6 | 0.50 | 50 | 0.17 |
|  | Ameland, Dutch Wadden sea | Amela | dune | 329 (2009) | 9 | 0.95 | 82 | 0.31 |
|  | Terschelling, Dutch Wadden sea | Tersc | dune | 1071 (2003) | 19 | 0.61 | 68 | 0.19 |
|  | Vlieland Kroonspolder, Vlieland, Dutch Wadden sea | Vliel | dune | 300 (2011) | 9 | 0.48 | 57 | 0.17 |
|  | De Weerribben | Dewee | fen | 1208 (2003) | 37 | 0.54 | 61 | 0.16 |
|  | De Wieden | Dewie | fen | 282 (2003) | 8 | 0.58 | 63 | 0.20 |
|  | Ankeveense plassen, Ankeveen | Ankev | fen | 116 (2003) | 5 | 0.55 | 50 | 0.15 |
|  | Het Hol, Loosdrecht | HetHo | fen | 855 (2003) | 9 | 0.72 | 69 | 0.25 |
|  | Nieuwkoopse plassen, Nieuwkoop | Nieuw | fen | 318 (2003) | 4 | 0.52 | 48 | 0.17 |
|  | Beerweg - Europort, Hoek van Holland | Beerw | dune | 160 (2009) | 10 | 0.35 | 36 | 0.13 |
|  | Oostvoornse meer, Voorne | Oostv | dune | 101 (2003) | 10 | 0.48 | 37 | 0.14 |
|  | Voornse Duin, Voorne | Voorn | dune | 100 (2003 | 6 | 0.54 | 55 | 0.16 |
|  | Hompelvoet, Grevelingenmeer | Hompe | dune | 70 (2009) | 17 | 0.76 | 69 | 0.22 |
|  | Veermansplaat, Grevelingenmeer | Veerm | dune | 20000 (2009) | 21 | 0.68 | 72 | 0.22 |
|  | Stampersplaat, Grevelingenmeer | Stamp | dune | 1600 (2009) | 14 | 0.65 | 71 | 0.23 |
|  | Dwars in de weg, Grevelingenmeer | Dwars | dune | 31 (2009) | 12 | 0.76 | 76 | 0.26 |
|  | Verklikkerduinen, Schouwen-Duiveland | Verkl | dune | 328 (2003) | 23 | 0.66 | 72 | 0.20 |
|  | Inlaag Hoofdplaat, Hoofdplaat | Inlaa | dune | 557 (2003) | 13 | 0.53 | 32 | 0.13 |
| Belgium | Hazop -Waaslandhaven, Verrebroek | Hazop | dune | 481(2009) | 30 | 0.65 | 42 | 0.16 |
|  | Meergoor, Mol | Meerg | fen | 5 (2009) | 5 | 0.93 | 69 | 0.24 |
| France | Dunes de Wulf | DeWulf | dune | 3 (2009) | 3 | 0.52 | 44 | 0.14 |
|  | Dunes du Slack, Ambleteuse | Slack | dune | 650 (2009) | 16 | 0.61 | 62 | 0.17 |
|  | Panne 11  Baie de la Canche, Camiers | Canch11 | dune | 800 (2009) | 5 | 0.45 | 45 | 0.13 |
|  | Panne 21-22  Baie de la Canche, Camiers | Canch21 | dune | 805 (2009) | 14 | 0.42 | 55 | 0.15 |
|  | Marais de Villiers, Villiers | Villi | fen | 42 (2009 | 18 | 0.56 | 37 | 0.14 |
|  | Camping des Dunes, Stella plage | Stell | dune | 3 (2009) | 3 | 0.56 | 46 | 0.16 |
|  | Panne 16  Merlimont | Merli16 | dune | 489 (2009) | 10 | 0.46 | 56 | 0.15 |
|  | Panne 18  Merlimont | Merli18 | dune | 4 (2009) | 3 | 0.78 | 56 | 0.27 |
|  | Blangy-Tronville | Blang | fen | 458 (2009) | 15 | 0.68 | 71 | 0.20 |
|  | Le Havre | LeHav | dune | 400 (2009) | 20 | 0.52 | 57 | 0.15 |
|  | Marais des Etelles,  La Tables des Etelles | Etell | fen | 65 (2009) | 15 | 0.64 | 69 | 0.20 |
| Hungary | Lake Velencei | Velen | fen | 3000 (2009) | 14 | 0.72 | 69 | 0.21 |
|  | Szigetcsép-up | Sziget | fen | 500 (2009) | 5 | 0.76 | 63 | 0.26 |
| Slovenia | Breg | Breg | fen | 20 (2009) | 3 | 1.03 | 61 | 0.30 |
|  | Crni potok | Crni | fen | 43.(2009) | 2 | 1.03 | 56 | 0.30 |
|  | Drenik | Dreni | fen | 42 (2009) | 3 | 1.4 | 73 | 0.35 |
|  | Sajevec | Sajev | fen | 35 (2009) | 2 | 0.96 | 53 | 0.26 |
|  | Trzin | Trzin | fen | 37 (2009) | 4 | 1.4 | 76 | 0.35 |

Nc: estimated census population size with year of observation between brackets, N: number of samples included in the AFLP-analysis, DW: frequency-down-weighted marker values, PPL: percentage of polymorphic loci at the 5% level and Hj: expected heterozygosity under F_IS_=0.83. ^1^: number of samples are cut back to a maximum of eight individuals for the calculation of PPL and H_j_. ^2^: calculated for F_IS_ = 0.83.
