# Supplementary material for: High levels of effective long-distance dispersal may blur ecotypic divergence in a rare terrestrial orchid
Source: BMC Ecol. 2014 Jul 7;14:20. doi: 10.1186/1472-6785-14-20 (PMC4099500; doi:10.1186/1472-6785-14-20)
Supplement: Additional file 2 — Number of DNA fragments generated by 4 AFLP primer-enzyme combinations used in Liparis loeselii. [file 1472-6785-14-20-S2.docx]

**Additional File 2**

**Number of DNA fragments generated by 4 AFLP primer-enzyme combinations used in *Liparis loeselii***

| Primer-enzyme combination | Scoring  range (bp) | cutRFU^1^ | Initial number of loci | Number of polymorphic loci with allele frequencies between  5% – 95% | Mean error rate ^2^(%) |
| --- | --- | --- | --- | --- | --- |
| *Eco*RI- ACT/*Mse*I-CAC | 80-600 | 100 | 716 | 67 | 2.9 |
| *Eco*RI-ACT/*Mse*I-CTA | 80-600 | 200 | 876 | 250 | 0 |
| *Eco*RI-ACT/*Mse*I-CTC | 80-350 | 100 | 480 | 78 | 3.53 |
| *Eco*RI-ACT/*Mse*I-CAA | 80-350 | 100 | 459 | 56 | 3.35 |
| *Total* |  |  | 2531 | 451 |  |
| *Mean* |  |  | 632.7 |  | 2.44 |

^1^ cutRFU: filter parameter in the scoring program RAWGENO [1] that eliminates bins with an average fragment fluor intensity below the parameter. The cutRFU parameter applies on normalized values.

^2^ Mean error rates were calculated in RAWGENO by summing differences between control profiles (i.e. samples that were genotyped twice) per primer-enzyme combination.

REFERENCE

1. Arrigo N, Tuszynski JW, Ehrich D, Gerdes T, Alvarez N: **Evaluating the impact of scoring parameters on the structure of intra-specific genetic variation using RawGeno, an R package for automating AFLP scoring**. *Bmc Bioinformatics* 2009, **10**.
