# Supplementary material for: High levels of effective long-distance dispersal may blur ecotypic divergence in a rare terrestrial orchid
Source: BMC Ecol. 2014 Jul 7;14:20. doi: 10.1186/1472-6785-14-20 (PMC4099500; doi:10.1186/1472-6785-14-20)
Supplement: Additional file 3 — Effect of different values of the minimal log likelihood difference (MLD) on the estimated rates of allocation success, of allocations to the wrong population and of non-allocations calculated with the simulation procedure of AFLPOP 1.1 and based on 1000 simulated genotypes and 10 iterations. [file 1472-6785-14-20-S3.docx]

**Additional File 3**

| **NORTHWEST FRANCE** | | | |  |  | | | |  | |  | | |  | |  |  |
| --- | --- | --- | --- | --- | --- | --- | --- | --- | --- | --- | --- | --- | --- | --- | --- | --- | --- |
| **Minimal log likelihood difference (MLD)** | **Average allocation success** | | | | | | | **Average rate of wrong allocations** | | | | **Average rate of**  **none-allocations** | | | | |  |
|  | Including all locations  (9 locations) | | Including locations with  n > 5  (6 locations) | | | | Including all locations  (9 locations) | | | Including locations with  n > 5  (6 locations) | | Including all locations  (9 locations) | | | Including locations with  n > 5  (6 locations) | | |
| 0 | 73.16 | 99.99 | | | | 26.72 | | | | 0.00 | | | 0.00 | | 0.00 | | |
| 1 | 71.59 | 99.98 | | | | 24.32 | | | | 0.00 | | | 4.08 | | 0.02 | | |
| 2 | 69.91 | 99.94 | | | | 21.97 | | | | 0.00 | | | 8.12 | | 0.06 | | |
| 3 | 68.61 | 99.86 | | | | 19.21 | | | | 0.00 | | | 12.17 | | 0.14 | | |
| **BELGIUM & THE NETHERLANDS** | | | | |  | | | |  | |  | | |  | |  |  |
| **Minimal log likelihood difference (MLD)** | **Average allocation success**  (16 locations) | | | | **Average rate of wrong allocations**  (16 locations) | | | | | | **Average rate of**  **none-allocations**  (16 locations) | | | | |  |  |
| 0 | 96.20 | | | | 3.80 | | | | | | 0.00 | | | | |  |  |
| 1 | 94.85 | | | | 2.54 | | | | | | 2.57 | | | | |  |  |
| 2 | 93.46 | | | | 1.61 | | | | | | 4.92 | | | | |  |  |
| 3 | 91.95 | | | | 0.92 | | | | | | 7.13 | | | | |  |  |
