# Supplementary material for: High levels of effective long-distance dispersal may blur ecotypic divergence in a rare terrestrial orchid
Source: BMC Ecol. 2014 Jul 7;14:20. doi: 10.1186/1472-6785-14-20 (PMC4099500; doi:10.1186/1472-6785-14-20)
Supplement: Additional file 4 — Re-allocation results of Liparis loeselii individuals performed with AFLPOP 1.1 for a minimal log likelihood difference (MLD) = 1. [file 1472-6785-14-20-S4.docx]

**Additional File 4**

**Re-allocation results of *Liparis loeselii* individuals performed with the program AFLPOP 1.1 [1] for a minimal log likelihood difference (MLD) = 1**

| **NORTHWEST FRANCE** |  | |  | | |  | |  |  |
| --- | --- | --- | --- | --- | --- | --- | --- | --- | --- |
| **number of polymorphic AFLP loci** | **449** | | **449** | | | **376** | |  |  |
| **number of putative source populations** | **9** | | **25** | | | **9** | |  |  |
| nb. of individuals sampled | 107 | | 107 | | | 107 | |  |  |
| nb. of individuals not allocated | 15 (14.0%) | | 17 (15.9%) | | | 18 (16.8%) | |  |  |
| nb. of individuals allocated to the sample population**^1^** (% of total allocated individuals) | 66 (71.7%) | | 50 (55.6%) | | | 64 (71.2%) | |  |  |
| nb. of immigrants^2^ allocated within sampled metapopulation | 24 | | 38 | | | 22 | |  |  |
| nb. of immigrants^2^ allocated outside sampled metapopulation | 2 (1.87%) | | 2 (1.87%) | | | 3 (2.80%) | |  |  |
| % immigrants^2^ from the total allocated individuals | 28.20% | | 44.40% | | | 28.00% | |  |  |
| nb. of. different source – destination combinations represented by the immigrants^2^ | 12 (13%) | | 21 (23%) | | | 16 (17.9%) | |  |  |
| Lower bound of estimated LDD (%) | 14.87% | | 24.87% | | |  | |  |  |
| **BELGIUM & THE NETHERLANDS** | |  | |  |  | | | |  |
| **number of polymorphic AFLP loci** | | **451** | | **451** | **378** | | | |  |
| **number of putative source populations** | | **16** | | **25** | **16** | | | |  |
| nb. of individuals sampled | | 224 | | 224 | 224 | | | |  |
| nb. of individuals not allocated | | 24 (10.7%) | | 33 (14.7%) | 40 (17.8%) | | | |  |
| nb. of individuals allocated to the sample population (% of total allocated individuals) | | 139 (69.5%) | | 129 (67.5%) | 133 (72.3%) | | | |  |
| nb. of immigrants^2^ allocated within sampled metapopulation | | 61 | | 62 | 51 | | | |  |
| nb. of immigrants^2^ allocated outside sampled metapopulation | | 0 | | 0 | 0 | | | |  |
| % immigrants^2^ from the total allocated individuals (upper bound of estimated LDD (%)) | | 30.50% | | 32.40% | 27.70% | | | |  |
| nb. of. different source – destination combinations represented by the immigrants^2^ (lower bound of estimated LDD (%)) | | 32 (16.5%) | | 33 (17.2%) | | | 29 (15.7%) | | |

**^1^**The nearby locations Merli16 and Merli18 were considered as one putative source population. ^2^putative immigrants detected by AFLPOP. This may also include offspring from immigrants produced by selfing.

REFERENCE

1. Duchesne P, Bernatchez L: **AFLPOP: a computer program for simulated and real population allocation, based on AFLP data**. *Molecular Ecology Notes* 2002, **2**(3):380-383.
