# Supplementary material for: High levels of effective long-distance dispersal may blur ecotypic divergence in a rare terrestrial orchid
Source: BMC Ecol. 2014 Jul 7;14:20. doi: 10.1186/1472-6785-14-20 (PMC4099500; doi:10.1186/1472-6785-14-20)
Supplement: Additional file 5 — Posterior probabilities for the model including selection of putative outlier loci detected with BAYESCAN 2.01 in pairwise population comparisons of Liparis loeselii. [file 1472-6785-14-20-S5.docx]

**Additional File 5**

**Posterior probabilities for the model including selection of putative outlier loci detected with BAYESCAN 2.01 in pairwise population comparisons of *Liparis loeselii*.**

|  | **Type of pairwise population comparison** | | |
| --- | --- | --- | --- |
| **Outlier locus ID** | **DUNE vs. DUNE** | **DUNE vs. FEN** | **FEN vs. FEN** |
| **12** |  |  |  |
| Hazop vs. Merli16 & Merli18 & Stell | 0.92 |  |  |
| **68** |  |  |  |
| Slack vs. LeHav | 0.97 |  |  |
| Slack vs. Villi |  | 0.98 |  |
| Tersc vs. Slack | 0.92 |  |  |
| Veerm vs. Slack | 0.96 |  |  |
| **80** |  |  |  |
| Slack vs. Villi |  | 0.98 |  |
| Hazop vs. Hompel | 0.95 |  |  |
| Hazop vs. LeHav | 0.99 |  |  |
| Hazop vs. Canch11 & Canch21 | 0.98 |  |  |
| Hazop vs. Merli16 & Merli18 & Stell | 0.99 |  |  |
| Hazop vs. Tersc | 0.99 |  |  |
| Hazop vs. Verkl | 0.99 |  |  |
| Hazop vs. Villi |  | 1.00 |  |
| **157** |  |  |  |
| Tersc vs. DeWee |  | 0.98 |  |
| **162** |  |  |  |
| Hazop vs. Canch11 & Canch21 | 0.97 |  |  |
| Veerm vs. DeWee |  | 0.99 |  |
| Verkl vs. DeWee |  | 0.97 |  |
| Verkl vs. Canch11 & Canch21 | 0.97 |  |  |
| **163** |  |  |  |
| Hompe vs. DeWee |  | 0.92 |  |
| LeHav vs. DeWee |  | 0.99 |  |
| Merli16 & Merli18 & Stell vs. DeWee |  | 0.91 |  |
| Tersc vs. DeWee |  | 0.98 |  |
| **164** |  |  |  |
| DeWee vs. Villi |  |  | 0.95 |
| Hompe vs. DeWee |  | 0.99 |  |
| Tersc vs. DeWee |  | 1.00 |  |
| Veerm vs. DeWee |  | 0.98 |  |
| **167** |  |  |  |
| DeWee vs. Blang |  |  | 0.99 |
| Slack vs Blang |  | 0.99 |  |
| Hazop vs. Blang |  | 1.00 |  |
| Hompe vs. Blang |  | 0.95 |  |
| LeHav vs. Blang |  | 0.96 |  |
| Canch11 & Canch21 vs. Blang |  | 0.99 |  |
| Merli16 & Merli18 & Stell vs. Blang |  | 0.98 |  |
| Tersc vs. Blang |  | 0.99 |  |
| Verkl vs. Blang |  | 0.99 |  |
| Veerm vs. Blang |  | 0.99 |  |
| **178** |  |  |  |
| DeWee vs. Villi |  |  | 0.95 |
| Slack vs. DeWee |  | 0.99 |  |
| Hazop vs. DeWee |  | 1.00 |  |
| Hompe vs. DeWee |  | 0.91 |  |
| LeHav vs. DeWee |  | 0.99 |  |
| Canch11 & Canch21 vs. DeWee |  | 0.99 |  |
| Merli16 & Merli18 & Stell vs. DeWee |  | 0.99 |  |
| Veerm vs. DeWee |  | 0.97 |  |
| **179** |  |  |  |
| DeWee vs. Villi |  |  | 1.00 |
| Slack vs. DeWee |  | 0.92 |  |
| Hazop vs. DeWee |  | 0.99 |  |
| Hompe vs. DeWee |  | 1.00 |  |
| Tersc vs. DeWee |  | 0.98 |  |
| Verkl vs. DeWee |  | 1.00 |  |
| **220** |  |  |  |
| HetHo, Nieuw & Ankev. vs. DeWee |  |  | 0.91 |
| HetHo, Nieuw and Ankev. vs. Verkl |  | 0.94 |  |
| **234** |  |  |  |
| DeWee vs. Blang |  |  | 0.99 |
| **283** |  |  |  |
| DeWee vs. Blang |  |  | 0.97 |
| **368** |  |  |  |
| Hazop vs. DeWee |  | 0.99 |  |
| **404** |  |  |  |
| Hompe vs. HetHo, Nieuw & Ankev. |  | 0.99 |  |
| Hompe vs. Villi |  | 0.99 |  |
| Veerm vs. Villi |  | 0.99 |  |
| Verkl vs. Villi |  | 1.00 |  |
| Canch11 & Canch21 vs. Villi |  | 0.90 |  |
| Tersc vs. HetHo, Nieuw & Ankev. | | 0.99 |  |
| Tersc vs. Blang |  | 0.99 |  |
| Tersc vs. LeHav |  | 0.99 |  |
| Veerm vs. HetHo, Nieuw & Ankev. |  | 0.99 |  |
| Verkl vs. HetHo, Nieuw & Ankev. |  | 0.99 |  |
| Verkl vs. Blang | 0.89 |  |  |
| Verkl vs. LeHav | 0.98 |  |  |
| **410** |  |  |  |
| Hazop vs. DeWee |  | 0.98 |  |
| Canch11 & Canch21 vs. DeWee | | 0.98 |  |
| **417** |  |  |  |
| Hazop vs. LeHav | 0.94 |  |  |
| **431** |  |  |  |
| Canch11 & Canch21 vs. HetHo, Nieuw & Ankev. | | 0.96 |  |
| Merli16 & Merli18 & Stell vs. DeWee |  | 0.98 |  |
| Tersc vs. DeWee |  | 0.95 |  |
| **439** |  |  |  |
| Hompe vs. Verkl | 0.97 |  |  |
| Veerm vs. Verkl | 0.99 |  |  |
| **440** |  |  |  |
| Veerm vs. Verkl |  | 0.98 |  |
| **444** |  |  |  |
| Hazop vs. Blang |  | 1.00 |  |
| Veerm vs. Verkl | 0.97 |  |  |
| Hompe vs. Blang |  | 0.99 |  |
| Hompe vs. Tersc | 0.99 |  |  |
| Hazop vs. Villi |  | 0.97 |  |
| Hazop vs. Villi |  | 0.98 |  |
| Verkl vs. Villi |  | 0.95 |  |
| Verkl vs. Blang |  | 0.99 |  |
| Verkl vs. Tersc | 0.99 |  |  |
| **446** |  |  |  |
| Hazop vs. Blang |  | 0.98 |  |
| Hompe vs. DeWee |  | 0.99 |  |
| Hazop vs. Merli16 & Merli18 | 0.91 |  |  |
| Hazop vs.Villi |  | 0.99 |  |
| Hompe vs. Villi |  | 0.90 |  |
| Verkl vs. Villi |  | 0.99 |  |
| Merli16 & Merli18 vs. Canch11 & Canch21 | 0.99 |  |  |
| Verkl vs. DeWee |  | 1.00 |  |
| Verkl vs. Blang |  | 0.93 |  |
| Merli16 & Merli18 vs. Verkl | 0.99 |  |  |
| Verkl vs. Tersc | 0.99 |  |  |
