# Supplementary material for: High levels of effective long-distance dispersal may blur ecotypic divergence in a rare terrestrial orchid
Source: BMC Ecol. 2014 Jul 7;14:20. doi: 10.1186/1472-6785-14-20 (PMC4099500; doi:10.1186/1472-6785-14-20)
Supplement: Additional file 6 — Detailed description on the search of putative outlier loci. [file 1472-6785-14-20-S6.docx]

**Additional File 6**

**Detailed description on the search of putative outlier loci.**

Outlier locus detection was performed based on 451 polymorphic loci by two commonly employed approaches implemented by the programs MCHEZA (Antao and Beaumont, 2011) and BAYESCAN 2.01 (Foll *et al*, 2008).

MCHEZA (Antao and Beaumont, 2011) is a selection detection workbench for dominant data based on the algorithm of the program DFDIST (Beaumont and Balding, 2004). This program uses a coalescent-based simulation approach to identify outlier loci displaying unusually high values of F_ST_ by comparing observed F_ST_-values with values expected under neutrality. We performed an initial run with 50000 simulations and all loci, using the mean neutral F_ST_ as a preliminary value. The function ‘Force mean F_ST_’ was chosen to simulate a more accurate estimate of the mean neutral F_ST_ by running a bisection algorithm over repeated simulations and by excluding all loci lying outside the 99% confidence interval, as their distribution could be the result of selection rather than neutral evolution. This refined estimate was used for a final set of 50000 simulations over all loci. To avoid a high rate of false positives among outlier markers due to multiple tests, we set the FDR to 0.05. The F_ST_ cut-off value for significant outlier detection was set to 0.99. The use of the trimmed mean F_ST_ and a low critical probability level (α = 0.01%) reduces the potential bias caused by AFLP size homoplasy in outlier detection (Caballero *et al*, 2008). For other parameter settings, we used the default option.

BAYESCAN 2.01 implements a reversible-jump MCMC algorithm to calculate posterior probabilities of two models: one including selection and one excluding selection (neutral). Departure from neutrality at a given locus is assumed when the locus-specific component (alpha) is necessary to explain the observed pattern of diversity (alpha significantly different from 0). A positive value of alpha suggests diversifying selection. The method provides posterior odds (PO) as the ratio of the posterior probability of the model of selection versus the neutral genetic model for each locus. In addition, the program allows for setting 'prior' odds for the two models. We used the default option that a neutral model was 10 times more likely than a model with selection. We ran ten pilot runs with a length of 5000 iterations. After an additional burn-in of 50000 iterations, we used 100000 iterations (sample size of 5000 and thinning interval of 20). According to Foll & Gaggiotti (2008), these parameters for the MCMC-algorithm ensure good convergence in most cases for binary data. To correct for multiple testing, we first defined a threshold of 5% for the FDR and then only considered loci with PO thresholds achieving this FDR using the R plot function provided along with BAYESCAN 2.01. From the latter loci, we defined an outlier as being significant using a threshold of PO >10 (or P(α≠0) > 0.91; i.e. ‘strong evidence’ for selection) for the rejection of the null hypothesis in each of the conducted tests (note that the final assembly of outlier loci was based on results from two algorithms on pooled samples and on the results of different population pairwise comparisons).

The power of BAYESCAN for detecting markers affected by selection is significantly reduced for comparisons including few samples (Foll *et al*, 2008). For the population pairwise comparisons, we therefore excluded populations with less than 15 sampled individuals unless they could be grouped with neighboring populations occupying the same habitat type. We pooled the samples of the populations with the population codes (Table 1): Merli16, Merli18 and Stell; Canch11 and Canch21; HetHo, Nieuw and Ankev. This resulted in a total of 36 fen-dune, 36 dune-dune and 6 fen-fen pairwise population comparisons.

**References**

Antao T, Beaumont MA (2011). Mcheza: a workbench to detect selection using dominant markers. *Bioinformatics* **27**(12)**:** 1717-1718.

Beaumont MA, Balding DJ (2004). Identifying adaptive genetic divergence among populations from genome scans. *Molecular Ecology* **13**(4)**:** 969-980.

Foll M, Beaumont MA, Gaggiotti O (2008). An approximate Bayesian computation approach to overcome biases that arise when using amplified fragment length polymorphism markers to study population structure. *Genetics* **179**(2)**:** 927-939.
